# The Assessment of the Integrated Antioxidant System of the Body in the Course of Radon Therapy: A Pilot Study

**DOI:** 10.1155/2018/6038106

**Published:** 2018-01-02

**Authors:** Jadwiga Kuciel-Lewandowska, Jan Gnus, Lilla Pawlik-Sobecka, Sylwia Płaczkowska, Izabela Kokot, Michał Kasperczak, Małgorzata Paprocka-Borowicz

**Affiliations:** ^1^Department of Physiotherapy, Medical University of Wroclaw, Wroclaw, Poland; ^2^Department of Professional Training in Clinical Chemistry, Medical University of Wroclaw, Wroclaw, Poland; ^3^Department of Diagnostics Laboratory for Teaching and Research, Medical University of Wroclaw, Wroclaw, Poland

## Abstract

**Introduction:**

The sources of Reactive Oxidative Species (ROS) in the organism are the respiratory processes occurring in cells catalyzed by different enzymes. Operation of ROS is balanced by antioxidants, the compounds; although present in low concentrations, they significantly inhibit the degree of oxidation of particular molecules.

**The Aim of the Study:**

The aim of this study was to assess the changes in the integrated antioxidant system under the influence of radon therapy in osteoarthritis patients.

**Material and Methods:**

Observation included 35 patients suffering from degenerative joints and disc disease (mean age 56.5 years) undergoing radon water therapy and control group that consisted of 15 osteoarthritis patients (mean age 54.2) without contact with radon water. Before therapy and after 18 days of treatment, serum total antioxidant status (TAS) was assessed with the use of standard colorimetric assay.

**Results:**

In the study group, we observed trends to increase TAS concentration, whereas, in the control group, TAS concentration was decreasing.

**Conclusions:**

(1) Radon waters treatment influenced the level of TAS of osteoarthritis patients treated with the radon water. (2) The change in TAS concentrations in the study group may be the result of low doses of ionizing radiation, but further studies on larger patient's groups are demanded. This study is registered with number NCT03274128.

## 1. Introduction

Reactive oxygen species (ROS) are a form of free radicals and include such molecules as singlet oxygen ^1^O_2_, superoxide anion O_2_^−^, hydrogen peroxide H_2_O_2_, and a hydroxide radical OH. In living organisms, the oxygen reacts with organic compounds oxidizing them without undergoing total, reduction due to the action of various, both external and internal, factors. A consequence of these shortcomings is formation of reactive oxygen species. In the state of health, the level of ROS is strictly controlled by maintaining a balance between their generation and removal [1–3]. Disturbance of this balance is defined as a shock or oxidative stress (OS). OS occurs either when the level of antioxidants, agents which reduce free radicals, is lowered or when, for various reasons, the generation of reactive oxygen species is intensified [4, 5]. As long as the balance between the generation and removal of ROS is maintained, they remain harmless to the cells and tissues. Impairment of this process leads to release of toxic ROS action which causes, among many other examples, the inflammatory diseases of the musculoskeletal system. In such case a chain of enzymatic reactions implicated in depolymerization of hyaluronic acid is started, leading to a loss of elasticity of the tissues, degradation of proteoglycans and collagen, protein oxidation, and the inhibition of chondrocyte proliferation [6]. ROS are associated with the pathogenesis other diseases such as atherosclerosis, neurodegenerative diseases, Alzheimer's disease, Parkinson's disease, various inflammations, allergies, cancer, diabetes, and macular degeneration. In the above-mentioned diseases, ROS affect both the transmission of cellular signals and activity of enzymes or genes involved in processes of proinflammatory factors, cell death, and DNA repair. Oxidatively modified cell membranes proteins lead to the reduction of its antioxidant properties, especially albumins, and predisposing to aggregation and deposition in tissues. The predominant effect of lipid peroxidation is the inhibition of cell membrane functions by inactivation of membrane enzymes and destroying its barrier properties [7, 8].

The human body has several mechanisms of both regulating and blocking the production of ROS. Antioxidant defense system includes the following:

1. Endogenous antioxidants, produced by the body:
  - Enzymatic-antioxidant enzymes: superoxide dismutase, glutathione peroxidase, catalase
  - Nonenzymatic: linolenic acid, polyamides, albumin, bilirubin, glutathione [9], uric acid, ceruloplasmin, transferrin, and coenzyme Q10
2. Exogenous antioxidants, delivered from the outside: vitamins C, A, and E, carotenoids, xanthophylls, and polyphenols [10–12]

The evaluation of the antioxidant system can be performed with the use of a variety of methods by assessing its individual parameters. A deficiency in any of these components can cause a reduction in the overall body antioxidant status. The total antioxidant status, especially created by nonenzymatic endogenous antioxidant compounds, could be measured globally by using commercial tests. Reduction in total antioxidant status has been implicated in many diseases, also mentioned above.

Radon water is specific water containing small quantities of unstable radioactive element, radon, and its decay products. The water is used in medicine, when the content of radon exceeds 74 Bq/l (2 nCi/l) and when it meets operational and hygienic requirements. Radon is commonly present, in trace amounts, in the spring and river waters, but for medicinal purposes it is obtained from natural outflows and wells. Radon is a chemical element formed in the disintegration of radioactive uranium and thorium. It is a noble, colorless, and odorless gas, soluble in water, especially weakly mineralized, or acidified. There are many radon isotopes, among which there is a precursor, radon-222 deriving directly from radium-226 through alpha decay. The emitted alpha particles have low penetrating power but high ionizing ability. They half-life is 3.8 days [13]. Radon in large doses has a negative impact on health and its adverse effects rely on damaging enzymes and nucleic acids, which leads to formation of neoplastic cells. Therefore, while using radon as a medicinal material one should have this in mind. The basis for rational therapeutic effect of radon water is the so-called hypothesis of radiation hormesis created in the 40s of the 20th century for pharmacology and toxicology. According to this theory, small doses of ionizing radiation activate life processes. The theory is similar to Arnd-Szulc principle used in physical therapy, founded in the nineteenth century, which says that weak doses of physical energy enhance physiological processes. Currently, there are no scientific reports explaining the mechanisms of radiation hormesis. Among the mechanisms, at the level of cell control system, there are stimulation of DNA repair processes, protein synthesis, activation of genes, production of stress proteins, detoxification of radicals, activation of membrane receptors, proliferation of splenocytes, and stimulation of the immune system, resulting in a lower risk of mutations or carcinogenesis. Changes occurring in the body are the result of ionization, which in living cells can be divided into the following stages:

1. Physical stage: energy being absorbed at the atomic level where there is an excitation and ionization of atoms or molecules
2. Physicochemical stage: the formation of radicals or radical-ions
3. Chemical stage: secondary reactions of radicals and ions with each other and with the environment
4. Biological stage: the reactions of living matter at different levels of the organization

The effects of radiation on living cells cause disturbances of various cell functions and depend on which kind of molecules was damaged. In case of somatic cells, huge role plays repair mechanisms, through which damaged molecules can be removed and replaced with a new molecule. This mechanism can be called the essence of the phenomenon of radiation hormesis [14, 15].

The concentration of radon in natural conditions changes constantly throughout the day, as well as seasonally, due to precipitation. In the course of therapy there is also observed radon loss for technical reasons: collecting water in reservoirs, transmission pipes, heating, chilling, and intense exploitation. The decrease of radon content varied from 40 to 80%. This large variation in the concentration of radon at a collecting place results in the fact that calculation of the dose is not possible; thus, it is not practiced [16].

Radon penetrates the skin in small quantities and the content is increasing at a higher temperature and humidity. The absorption of radon in 95% occurs in lungs, whereas excretion from the body occurs at 90% also through the lungs and the remaining part through kidneys and skin. During baths absorption occurs mainly through inhalation, because radon and its derivatives accumulate in large quantities above the water. The lungs are exposed to radon, in significant extent, due to the deposition of the degradation products in the alveoli. The radioactive residues are deposited on the skin surface and remain there for several hours. Radon radioactive decay in the body is very diverse and largely depends on the amount of body fat, but adrenal cortex, liver, and muscles also play an important role in this process. Anti-inflammatory, desensitizing, and analgesic action of radon can be explained by the stimulation of the adrenal cortex and increased production of steroid hormones. Baths in the radon water affect hormonal regulation in both women and men. This process is followed by increased activity of the endocrine glands persisting for about 3 months. An increase in concentrations of luteinizing hormone and growth hormone in serum, accompanied with an increase in cortisol, testosterone, estradiol, and estriol in serum was observed after radon baths. In women in menopause period, elevated level of estradiol and follicular maturation abolish the symptoms of menopause, whereas in men improvement of semen quality and increase of sperm count and motility were observed. Radon treatments have influences on improving peripheral circulation, reducing swelling, arthritis, tendon, and muscle pain and improve performance mobility. There were also such processes observed as lowering of blood pressure, cholesterol, and triglycerides concentration, decreased red blood cell sedimentation rate and increased hemoglobin concentration and red blood cells count, increasing levels of ionized calcium, parathyroid hormone, and calcitonin, and speeding up the removal of harmful products of metabolism [17–20].

The imbalance in oxidative status is well known in osteoarthritis patients. Using a radon waters adjunct 21-day treatment is established in many countries which possess such kinds of balneotherapy sources.

In this context, it could be interesting to assess the influence of radon baths on antioxidant status in osteoarthritis patients, as a one of potential mechanisms of curative action of balneotherapy.

## 2. Aim of the Work

The aim of the study was to evaluate the changes in the antioxidant system of the integrated system under the influence of radon therapy in osteoarthritis patients.

## 3. Material and Methods

### 3.1. Studied Group

The studies were nonrandomized. The study group consisted of *n* = 35 patients with joints and spine pains caused by osteoarthritis or a discectomy. The age of patients ranged 47–63 years with mean 56.5 years. Among the respondents there were 24 women and 11 men. The essential criterion for the selection of patients was the presence of degenerative joints and/or disc disease, age range 45–65 years, the written consent to participate in research and no impediment to comprehensive treatment at the spa. The exclusion criteria were the lack of consent to participate in research, age under 45 and over 65 years, the presence of diseases constituting a contraindication to therapy (compatible with standard list of indications and contraindications to spa therapy), and the presence of metabolic diseases. The patients were on normal or light diet, dominated by dishes prepared with low-fat content. Both diets were standard calories diets and there were no vitamin supplements used. The study was designed with participation of the control group consisted of 15 people selected from the employees of the spa, including 9 women and 6 men, aged from 50 to 62 years, mean 54.2. The control group embraced patients suffering from osteoarthritis of the musculoskeletal system, not using treatments at the spa and had no direct contact with radon waters. In both the control and the study group, there were the same inclusion and exclusion criteria for participation in the tests.

### 3.2. Study Design

The study was approved by the Bioethics Committee of the Medical University in Wroclaw, Opinion number 135/2015; also written consent from the President Swieradów-Czerniawa Spa and individual written consent of the patients prepared in accordance with the model recommended by the Bioethical Committee of the Medical University in Wroclaw were obtained. The documentation is held by the authors of the work. The study was conducted in the health resort Świeradów-Zdroj, Poland. The observation included patients undergoing treatment at the spa within the 21-day curative stays. The conducted study concerns curative radon water from Świeradów-zdroj that has been used in treatments for more than one hundred years. The natural water with low mineralized content plays a major therapeutic role with the parameters of Rn 303,1–441,5 Bq/l. In the treatment rooms (inhalation, cabins with baths, and swimming pool), measurement of alpha radiation was 184,4–450,0 MeV. This evaluation allows one to determine the exposure of the patient. In contrast, the calculation of absorbed dose of radiation is not carried out because it is a variable value. It depends both on the body anatomy, especially fat content and surface of absorption, and on presence of some diseases, and radon loss dependent on its exploitation as mentioned in Introduction. Measurements were performed in room on daily basis with the use of certified detectors. The measurements were analyzed every 3 months at the Institute of Occupational Medicine in the Department of Radiation Protection in Lodz, Poland. Among the types of treatments used in the therapy, there was comprehensive radon bath, with the temperature of 37°C, duration 15 min.; the treatments were performed every 2nd day, whereas, for radon inhalations that lasted 15 min. with the temp. 37°C, treatments were performed every 2nd day from Monday to Friday. Baths and inhalations were performed interchangeably and the total number of radon treatments during one stay was 15. In addition, the following forms of therapy was used: kinesiotherapy, duration of 30–45 min; physiotherapy, in limited extent due to the possibility of activating oxidation processes. Model of radon therapy in Europe has a tradition lasting over a hundred years. Naturally, therapeutic programs were modified with actively developing hydrotherapy and balneotherapy.

### 3.3. TAS Measurement

Venous blood was collected from patients under fasting condition, before and after 18 days of treatment at the spa. To evaluate the total antioxidant status in heparin plasma, a commercial colorimetric assay was applied (Randox Laboratories, Ltd.) and performed on chemical analyzer Konelab 20i (Thermo Scientific).

### 3.4. Statistical Analysis

Statistical analysis was performed with using Statistica 12, PL application. For the measurable variables, there were the arithmetic means, standard deviations, and range of variability (extreme values) calculated. All tested variables quantitative variables were analyzed with the use of Shapiro-Wilk's test to determine the type of distribution. The comparison between the results of measurements in each group was performed using a post hoc Friedman ANOVA test. For all comparisons. the level of *α* = 0.05 was assumed and the obtained values were rounded to three decimal points.

## 4. Results

**Figure 1:**
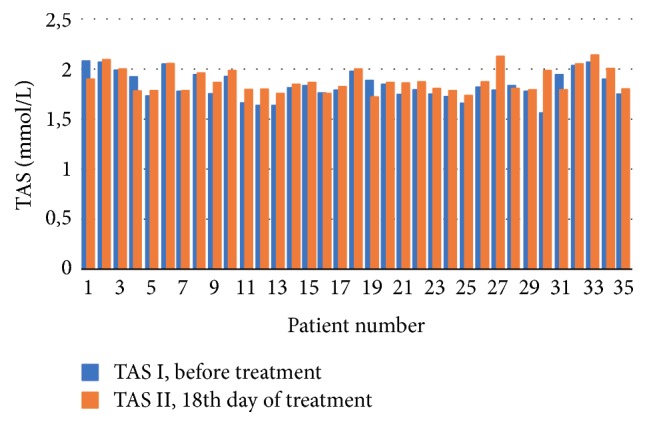
Study group: the obtained TAS concentration levels before and at the 18th day of treatment.

**Figure 2:**
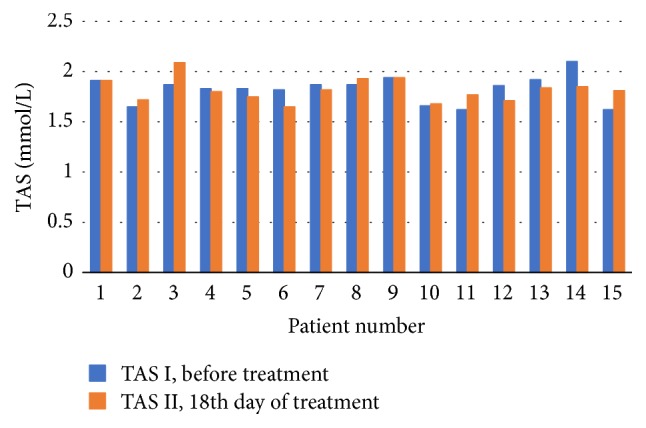
Control group: the obtained TAS concentration levels before and at the 18th day of treatment.

Figures 1 and 2 provide distribution of TAS concentrations values in each of the patients the at the first (TAS I) and second (TAS II) study point in both groups.

**Figure 3:**
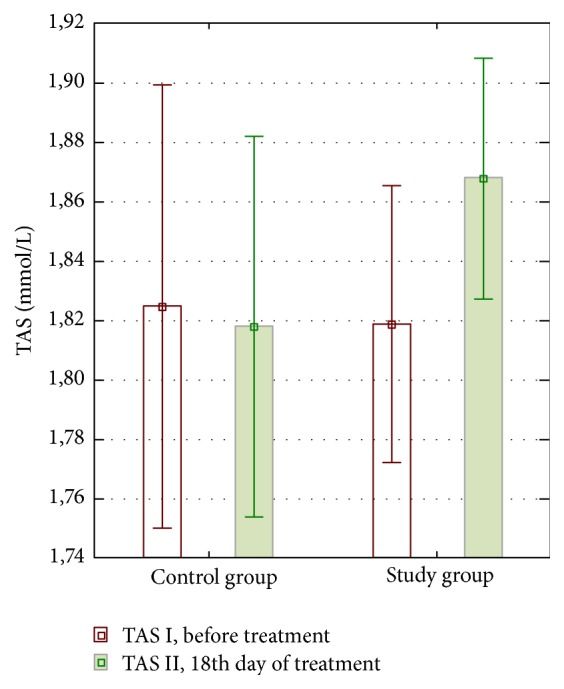
Distribution of TAS mean concentrations obtained in both groups at the first (TAS I) and second (TAS II) study point. The data are presented as mean and 95% Confidence Interval for each of the proper groups.

In the study group, an increase in the level of TAS in the 18th day of treatment was demonstrated. In the control group, in the same study point, TAS level dropped below the output value. The *p* value in the comparison measurement before and in 18th day of treatment for both groups did not reach the assumed level of significance. Both the increase in value in the first group and the decline in value were not significant. The data was shown in Table 1 and Figure 3.

## 5. Discussion

Radon therapy in Polish conditions can be carried out only on medical commission after considering the indications and contraindications, in fixed standard dose. The number of treatments, their duration, and type are specified, such as inhalations, baths, or mouthwash, especially of periodontium. Thermal spas use water from natural sources from the drilling performed in accordance with the mining law, with the approval of the Ministry of Environmental Protection and under the supervision of the Ministry of Health and Social Welfare. Medicinal water must meet certain criteria of chemical and bacteriological testing to be considered as curative.

In our study, we observed an increase of total antioxidant status in the study group, whereas the control group showed a decrease in total antioxidant capacity of the biological system. In both cases, the occurring changes were not statistically significant. Preliminary tests were performed with the assumption of further increase in the number of groups participants and monitoring the changes in the age groups considering the sex of the participants. In the conducted tests the participants from the control group did not take part in the radon treatments; thus it can be assumed that because of this reason there was no increase in TAS level. The question concerning the decrease has no clear answer. There can be many reasons for such status: a change of diet, drugs, stimulants, stress, excessive exercises, and others. However, the obtained results indicate the sensitivity of the antioxidant system to the spa physiotherapy, although in the study group the changes in the concentration of TAS were not significant but more visible in comparison to control group. The reason for this result could be a small number of participants in the group and smaller chemical activity of radon water resulting from poor mineralization (low levels of metabolically active elements and their compounds, less than 1000 mg dissolved in 1 liter of water). In case of medicinal waters in Świeradów, however, the most crucial element was the presence of ionizing radiation. Biological effect of ionizing radiation depends on the conditions of irradiation: dose rate, a fractionation method, the masses of irradiated tissue, the type of irradiated organs, tissue oxygenation, and biological characteristics of the system. With low power radiation, the reparative capability of the body is higher. The body tolerates better doses distributed to several fractions, at appropriate intervals. This method of applying small doses of ionizing radiation is used in radon spa therapy, when the body gets the time needed to repair the damage of radiation in a natural way. Irradiation of large amount of tissues in small doses brings better systemic effects. A possible positive effect is the increase in the body's immunity due to not very well known mechanisms of transitional functional or morphological changes dependent on the stimulation of the autonomic nervous system. This mechanism may give rise to positive changes determining systemic improvement after the spa treatment [21].

Activation of the antioxidant system may affect many metabolic changes, which are discussed in Introduction. It is difficult to assess the extent to which ionizing radiation stimulates the system. There are not many publications concerning the results of the carried out observations. Medical journals database was searched. Most of the articles developed in the 60s, 70s, and 80s present discussions on the effects of radon therapy in general terms without control groups. There were a few randomized trials conducted on not very large groups. What is more, there were no mechanisms of these changes distinguished. Radon water was considered as the main factor causing systemic change and the reduction or disappearance of pain. A recent study conducted in Japan on laboratory animals subjected to inhalation of radon shows the assessment of the impact on the respiratory system and the consequent systemic transformation. These results seem similar to the results from the past [22, 23]. Studies conducted in the 90s by Reinisch showed that, under the influence of radon, there was decrease of ROS release from neutrophils in patients with ankylosing spondylitis [24].

It was also shown that small doses of radon stimulate the activity of some enzymes, for instance, superoxide dismutase [25]. A publication on the impact of the carbonic acid tub-bath on the transformation of selected elements of antioxidant system was also found. There were three enzymes marked belonging to the antioxidant system, that is, superoxide (SOD), catalase (CAT), glutathione peroxidase (GSH Px), and marker of malondialdehyde lipid peroxidation (MDA). The study was performed on each enrolled patient three times, that is, before therapy, one hour after the first treatment and 24 hours after the last of the ten treatments. Before and after the therapy, total cholesterol and its fractions were determined. After applying a series of balneotherapy treatments, there was no change in the activity of SOD, CAT, and GSH Px and MDA concentrations found but there was a trend toward the improvement of the lipid profile observed [26].

In view of the importance of ROS transformations in the development of pathologies, it can be assumed that the beneficial effect of radon therapy may be caused by increase in total antioxidant capacity of the system. Radon, which is also a source of free radicals, stimulates this system. By stimulating of both multiple metabolic pathways and the endocrine system, the body achieves metabolic balance and increases defense capacity. However, huge doubts, in many scientific circles, are raised by the stimulation of the system with low doses of ionizing radiation. The reason may be the lack of a full evaluation of the phenomenon of radiation hormesis, which is considered a positive phenomenon causing the body's repair processes [27, 28]. Undoubtedly, there is no research in line with the principles of evidence-based medicine regarding radon spa therapy. This is due to inability to perform blind trials conducted in spa therapy, setting of absorbed dose radiation, and doubts regarding the value of the whole therapy. Despite the difficulties, it is necessary to undertake the research. Positive clinical effects of the therapy encourage further testing. Moreover, in regions where radon water is present lower morbidity and lower number of deaths from cancer were observed than in the neighboring communities. There were many other publications released depicting positive health effects of exposure to ionizing radiation. However, the above-mentioned reports were related to occupational exposures, the population living in areas of a nuclear explosion, and studies of the population living in areas with high natural background radiation. All these studies show that low doses of radiation have a neutral or even a positive impact on health [29, 30].

From clinical observations, we get to know that spa medicine obtaining positive effects of therapy depends on the type and intensity of the applied stimuli, reactivity of the system, individual susceptibility, presence of other diseases, and genetically determined enzymatic systems. A particular difficulty in therapeutic treatment lies in the use of natural medicinal resources, for example, healing waters due to small pharmacodynamic stability. Due to the metabolic activity of chemical compounds contained in the therapeutic waters, the basis of a good treatment should be individually chosen and leading to therapy. In the case of radon water therapy, there are big problems such as the absorbed dose of radiation, the scope of changes in the system, and the loss of radiation intensity during distribution of radon water. Can the use of radon water for decades and the positive reception of this therapy by the patients already constitute proof of its effectiveness? In view of the complexity of the spa treatment, it is difficult to emphasize particular effectiveness of radon water therapy. The final result of spa treatment is always the sum of the actions of various forms of therapy. Assessment of positive impact of radon therapy on the human body requires conducting multidisciplinary research with a specific explanation of the essence of the phenomenon of hormesis. It is imperative to design randomized study conducted on large groups.

## 6. Conclusions

1. The trend to increase in the level of antioxidant status in the body of osteoarthritis patients treated by radon waters in the spa was demonstrated.
2. The change in TAS concentrations in the study group may be the result of low doses of ionizing radiation originating from radon dissolved in medicinal water used in therapy, but further studies on larger patient's groups are demanded.

## Figures and Tables

**Table 1:** The concentration of TAS values in mmol/L for both groups.

	*N*	$\overset{-}{x}$	Min	Max	SD	Multiple comparisons *p* ^*∗∗*^ value
Study group						
TAS I	35	1,82	1,55	2,06	0,14	TAS I versus TAS II *p* = 0,379
TAS II	35	1,87	1,71	2,13	0,12	
Control group						
TAS I	15	1,83	1,65	2,09	0,11	TAS I versus TAS II *p* = 0,944
TAS II	15	1,81	1,62	2,10	0,14	

$\overset{-}{x}\;\;$–mean; Min, minimum; Max, maximum; SD, standard deviation; ^*∗∗*^multiple comparisons in post hoc ANOVA Friedman test; TAS I, first measurement; TAS II, second measurement.
